# The incidence of HIV and associated risk factors among pregnant women in Kabarole District, Uganda

**DOI:** 10.1371/journal.pone.0234174

**Published:** 2020-06-05

**Authors:** Hannah Schumann, Kenyonyozi Rubagumya, John Rubaihayo, Gundel Harms, Rhoda K. Wanyenze, Stefanie Theuring

**Affiliations:** 1 Institute of Tropical Medicine and International Health, Charité–Universitätsmedizin Berlin, corporate member of Freie Universität Berlin, Humboldt-Universität zu Berlin and Berlin Institute of Health, Berlin, Germany; 2 School of Health Sciences, Mountains of the Moon University, Fort Portal, Uganda; 3 School of Public Health, Makerere University, Kampala, Uganda; African Population and Health Research Center, KENYA

## Abstract

**Objectives:**

The study attempted to determine the incidence of HIV among pregnant women in Kabarole District, Uganda, and to identify socio-demographic and behavioral risk factors for seroconversion during pregnancy.

**Methods:**

We carried out a retrospective cohort study among women for whom a documented HIV-negative test result from the first pregnancy trimester could be confirmed using available records, and who were HIV-retested in the third trimester or during delivery. In total, 1610 pregnant women from three different healthcare settings took part in the study. We captured the results of repeated HIV tests and conducted semi-structured interviews to explore participants’ socio-demographic characteristics and sexual risk behavior. For HIV incidence rates, we calculated the number of seroconversions per 100 person-years. We used Fisher's exact test to test for potential associations. Penalized maximum likelihood logistic regression and Poisson regression were applied to adjust for potential confounders.

**Results:**

The overall HIV incidence rate among participants was 2.9/100 women-years. Among socio-demographic characteristics, the multivariable analysis showed a significant association of marital status with HIV incidence in pregnancy (IRR 8.78, 95%CI [1.13–68.33]). Risky sexual behaviors including higher number of sexual partners in pregnancy (IRR 2.78 [1.30–5.94]), unprotected sex with unknown persons (IRR 14.25 [4.52–44.93]), alcohol abuse (IRR 12.08 [4.18–34.90]) and sex under the influence of drugs or alcohol (IRR 6.33 [1.36–29.49]) were significantly associated with seroconversion in pregnancy (similar results in logistic regression).

**Conclusions:**

HIV incidence was three times higher among our pregnant study population compared to the general female population in Uganda. This underlines the importance of HIV prevention and repeat testing during pregnancy. Identified risk groups should be considered for pre-exposure prophylaxis.

## Introduction

Despite substantial worldwide success in the diagnosis and treatment of HIV and AIDS, new infections remain a considerable challenge. UNAIDS portrayed the situation in 2018 as a prevention crisis [1]. Studies have shown that around 60% of new adult HIV infections in Sub-Saharan Africa occur in women; this gender disparity is especially pronounced among adolescents and young adults, where the incidence of HIV among females is up to eight times higher than among males [1, 2].

The prevention of seroconversion in pregnancy is particularly important among this at-risk population. Excessive levels of viral load, which occur during primary infection, represent the most significant risk factor for mother-to-child transmission. Therefore, pre- and perinatally acquired maternal HIV infections contribute considerably to the overall paediatric burden of HIV, accounting for around one third of new infections in children [3, 4].

Several studies have been conducted to determine HIV incidence during pregnancy; a meta-analysis of these studies showed a pooled rate of 4.7/100 person-years. The risk of acquiring HIV was significantly higher among pregnant women in the African countries. In the meta-analysis, incidence estimates in three assessed studies from Uganda were below the average HIV incidence estimate among pregnant women in Sub-Saharan Africa [5].

Although pregnant women appear to have a higher incidence of HIV compared to non-pregnant women, the evidence is rather ambiguous when it comes to the effect of pregnancy on HIV acquisition [6]. Some studies have suggested pregnancy may have a protective effect, attributing the reported higher HIV incidence among pregnant women to other confounding elements including behavioral factors typically associated with youth, such as being more sexually active and having male partners with more external sexual relationships. Other studies have suggested that pregnancy may impose a risk for HIV acquisition because of pregnancy-related immunological or hormonal changes, including elevated estrogen and progesterone levels leading to increased ectopy (hyperplasia of the columnar epithelium, hyperemia and stromal edema), resulting in elevated susceptibility to HIV among pregnant women [6–8]. Although the general risk factors for HIV seroconversion are known, it is not yet clear which of them are specifically relevant to pregnant women.

Uganda is among the parts of the world that have been burdened most by HIV; there are 1.3 million people living with HIV in the country. The annual number of new infections has decreased from 160.000 in 2010 to 83.500 in 2015. The overall incidence rate in Uganda was 0.76% in 2014 and is estimated to go down to 0.46% by 2020 [9]. Services for prevention of mother-to-child transmission (PMTCT) are well established in the country, and antiretroviral drug coverage has significantly expanded and now includes 95% of the pregnant women living with HIV, while in 2010 PMTCT coverage was only 31% [1,9]. However, there seems to be a paucity of literature on the incidence of HIV infection during pregnancy in Uganda. The last study to be conducted on this topic was a prospective study carried out in 2005 in Rakai District [6]. This study showed a higher risk of HIV acquisition among pregnant women, suggesting the need for preventative measures during pregnancy in order to protect mothers and their babies. Nonetheless, there has not been a study of rates of new HIV infection in Uganda since then. As a result there are substantial unanswered questions regarding both the HIV incidence in the country in general and more specifically with regards to risk factors that are particularly relevant during pregnancy. The objectives of our study were therefore to determine the incidence of HIV seroconversion among pregnant women in Uganda’s Kabarole District, and to identify socio-demographic, health-related and behavioral risk factors for contracting HIV during pregnancy. Fort Portal municipality in Kabarole District has been one of the most severely HIV-affected communities in Uganda with prevalence rates as high as 16%, and represents our study setting [10].

## Methods

### Study design, population and settings

A retrospective cohort study was conducted in 2017 in Fort Portal, Kabarole District, Uganda. The district has a population of around 470.000. The capital, Fort Portal, has around 50.000 inhabitants [11]. A 2010 study estimated HIV prevalence among pregnant women in the district at about 10% [12]. Our research was designed as a multicentre study using a maximum diversity approach, for which three hospitals were purposively selected as study sites. These hospitals represented three different healthcare settings: public rural (Kibiito Health Center IV), public urban (Fort Portal Regional Referral Hospital, Buhinga) and a private urban catholic hospital (Holy Family Virika Hospital). Each of them offered free standard antenatal care (ANC), obstetric and postpartum services, as well as HIV care. Women were tested for HIV at their first ANC visit as a routine procedure at each of these health care facilities. All three study sites follow the national testing algorithm using Determine, Stat-pak and SD-Bioline [13]. If the women tested positive, they were introduced into existing PMTCT structures, and started Option B+ (lifelong ARV treatment for all pregnant women tested HIV positive and prophylactic ARV treatment for all infants born to HIV positive mothers for 4–6 weeks after birth) [14]. If the primary HIV test at the first ANC visit was negative, or a negative HIV test result from another facility was documented in their ANC card, women were retested in the third trimester (28–36 weeks of gestational age) as recommended in the national protocol. Those women not retested in the third trimester for any reason were offered a repeat test upon delivery. In all three healthcare settings, apart from repeat testing, there were no further specific activities in place regarding HIV prevention in HIV-negative pregnant women.

### Eligibility criteria

Any woman who met the following criteria was considered eligible for recruitment in the study: She was an ANC client who visited one of the health facilities in her third trimester, or a delivery client in the maternity ward; she had been tested and was HIV-negative in the first or second trimester of pregnancy, and this was clearly documented in her ANC card; she was at least 15 years old (mature minor); and she was willing to give written informed consent to participate in the study. Exclusion criteria were age below 15 years, and any clinical condition disturbing the ability of the client to give informed consent and to participate, including mental illness or an acute health condition.

### Recruitment and data collection

Recruitment of participants and data collection took place in the ANC clinic and maternity wards of all three hospitals from June to December 2017. After informed consent of eligible clients, rapid repeat tests for HIV were conducted as part of the routine hospital procedures and in compliance with the national and WHO standards. Results were disclosed to the participants during post-test counselling. If a participant had seroconverted, extensive post-test and psychosocial counselling took place before the study interview was conducted.

For the study, the date of the negative HIV test earlier in pregnancy was transferred from the ANC card to a study form, where the date and result of the repeat HIV test was also noted. After noting the test result, a trained study nurse conducted a structured interview with the client in a confidential atmosphere. The interview was based on a questionnaire developed specifically for the study, and which had been pilot tested in a subgroup of ten clients per facility and adjusted where necessary prior to data collection. It focussed on potential socio-demographic, health-related and behavioral risk factors presumed to be associated with HIV infections in accordance with pre-existing literature (the questionnaire is available in the S1 Data). Those variables included maternal factors like age, marital status, number of children, and presence of sexually transmitted diseases, as well as paternal factors such as the partner´s occupation, HIV status, health seeking behavior, circumcision; and potential risk behaviors of the woman or her partner, such as number of sexual partners, use of condoms, alcohol abuse or intimate partner violence. Women were also asked to self-assess their perceived risk for obtaining HIV infection in four categories from “high risk” to “not at risk at all”. In order to evaluate their socio-economic status (SES), participants were asked about the availability of certain items within the household, including radio, fridge, a motorbike or car, electricity, tap water, a cupboard, TV, cattle and a mosquito net. Each item was given one point upon presence, resulting in an unvalidated wealth score ranging from zero to nine.

### Statistical analysis

The primary data set was entered into Excel anonymously and cleared of double entries. Statistical analysis was conducted using Stata 13.0 software (Stata, Texas, USA).

The incidence rate was calculated by number of seroconversions in pregnant women per 100 person-years, based on the intervals between the last negative HIV test to the time of the follow-up HIV test during the third trimester or the postpartum period. Differing HIV incidence rates were presented for all sub-groups based on the socio-demographic, health-related and behavioral risk factors. Fisher's exact test was chosen to test for potential associations, taking into account the low HIV incidence in the study population.

In order to adjust for potential confounders, two regression models were used to test for the association of socio-demographic characteristics of the participants with HIV incidence in pregnancy: we employed penalized maximum likelihood logistic regression, which is tailored to analyze rare events in binary variable data, and Poisson regression, which is tailored for comparing rates in rare events. Furthermore, two tests of goodness of fit after Poisson were conducted, Deviance goodness of fit test and Pearson goodness of fit test. Both presented insignificant P-values of 1.000 and 0.099 respectively, suggesting the model to be a good fit. For both regression models used in the study, observations with missing values were excluded for all categorical or binary variables.

The two models were also used to test for the association of sexual risk behaviors with HIV incidence during pregnancy. Both regression models were adjusted for five socio-demographic characteristics including: age, marital status, education, wealth score and partner HIV status.

### Ethical considerations

The study protocol was approved by the Makerere University School of Public Health Higher Degrees Research and Ethics Committee, by the National Council for Science and Technology in Uganda, and by the Ethics Committee, Charité—Universitätsmedizin Berlin, Germany. All data was treated highly confidential and was only accessible in password-protected files for authorized study staff.

## Results

### Socio-demographic characteristics of the study population

Most participants were adolescents or young adults below 24 years of age, the mean age was 24.7 (SD 5.86). Almost half of the women had primary education and one third had secondary education, in contrast, only five per cent did not receive any education. With regard to parity, one fifth were having their first child, while one forth had already three or more children. Nearly 40% of the women were married or living together with their partner, whereas almost half were in a relationship, but living separately. Concerning their SES, the participants showed a mean wealth score of 4.4 on a scale of zero to nine.

Around 80% of the male partners were 25 years of age or older. The intra-couple age difference was six years on average. One third of the male partners had primary education while 60% had secondary or tertiary education. Participants were asked to report on the HIV status of their partners. 79% were reported as known negative and only 3% were described as known positive or known and not specified, while 18% of the women did not know their partner’s HIV status. In total, almost 75% percent of the women were recruited from an urban environment (Table 1).

**Table 1:** The socio-demographic characteristics of the study participants.

Variable	Mean (SD) or Median (Range)	Number	Percentage (%)*
**Age**	**24.7 (5.86) Mean (SD)**	**1604**	**100.0**
15–24 years of age		893	55.7
25–34 years of age		580	36.2
35–50 years of age		131	8.2
**Facility**		**1610**	**100.0**
Buhinga (urban)		703	43.7
Kibiito (rural)		408	25.3
Virika (private urban)		499	31.0
**Gravidity**	**1 (1–18)**	**1095**	**100.0**
One	**Median (Range)**	319	29.1
Two		270	24.7
Three		205	18.7
Four or more		301	27.5
**Parity**	**1 (0–13)**	**1503**	**100.0**
None	**Median (Range)**	314	20.9
One		489	32.5
Two		296	19.7
Three or more		404	26.9
**Marital status**		**1608**	**100.0**
Married or cohabiting		625	38.9
None cohabiting couple		754	46.9
Single, widowed or divorced		229	14.2
**Education**		**1606**	**100.0**
None		87	5.4
Primary		761	47.4
Secondary		553	34.4
Tertiary		205	12.8
**Religion**		**1610**	**100.0**
Christian		1434	89.1
Muslim		79	4.9
Other		97	6.0
**Occupation**		**1598**	**100.0**
Housewife		437	27.4
Farmer		564	35.3
Trader		282	17.7
Civil servant		165	10.3
Other		150	9.4
**Wealth score**	**4.4 (0.05)**	**1610**	**100.0**
Low SES 0–3	Mean (SD)	574	35.7
High SES 4–9		1036	64.4
**Travel distance in minutes**	**30.2 (0.43)**	**1463**	**100.0**
Less than or equal to 30 minutes	**Mean (SD)**	1038	71.0
More than 30 minutes		425	29.1
**Partner age**	**30.6 (0.19)**	**1597**	**100.0**
14–24 years of age	**Mean (SD)**	341	21.4
25–34 years of age		794	49.7
35–60 years of age		462	28.9
**Partner education**		**1590**	**100.0**
None		56	3.5
Primary		587	36.9
Secondary		677	42.6
Tertiary		270	17.0
**Partner occupation**		**1602**	**100.0**
Farmer		473	29.5
Trader		430	26.8
Civil servant		191	11.9
Driver		152	9.5
Armed forces		47	2.9
Trucker		30	1.9
Other		279	17.4
**Partner HIV status**		**1607**	**100.0**
Known negative		1275	79.3
Known positive		20	1.2
Known and not specified		23	1.4
Unknown		289	18.0

* Valid percentage: observations with missing values were excluded.

SD, Standard deviation.

### HIV incidence rate during pregnancy

Fifteen out of 1610 women showed HIV seroconversion during the study period, which accounts for an incidence rate of 2.85/100 person-years. The average time between HIV tests was 17 weeks. The total time of follow up of the participants summed up to 526 person-years. The three hospitals included in the study (urban, rural, private urban) varied to a great extent in HIV incidences (3.01, 5.16 and 0.64/100 person-years, respectively). However, the difference between the sites was revealed to be merely of borderline significance (p-value 0.052, overlapping confidence intervals).

### Association of socio-demographic characteristics with HIV incidence during pregnancy

Univariable analysis using Fisher’s exact test (Table 2) showed only marital status, women’s education and SES to have significant associations with HIV incidence during pregnancy (p-value 0.018, 0.008 and 0.015 respectively).

**Table 2:** HIV incidence rate during pregnancy (in 100 women-years).

Variable	Number of HIV seroconversion (person-years)	HIV incidence rate (95% Confidence Interval)	P-value Fisher's exact test
Total study population (1610)	15 (525.5)	2.9 (1.72–4.73)	-
Age group (n = 1604)			
15–24 years of age	10 (296.0)	3.4 (1.82–6.28)	0.671
25–34 years of age	5 (186.1)	2.7 (1.12–6.45)	
35–50 years of age	0 (41.3)	0 (-)	
Facility (n = 1610)			
Buhinga (public urban)	7 (232.6)	3.0 (1.44–6.31)	0.052
Kibiito (public rural)	7 (135.6)	5.2 (2.46–10.83)	
Virika (private urban)	1 (157.4)	0.6 (0.09–4.51)	
Gravidity (n = 1095)			
One	3 (103.5)	2.9 (0.94–8.98)	0.738
Two	3 (87.1)	3.4 (1.11–10.68)	
Three	1 (64.0)	1.6 (0.22–11.09)	
Four or more	1 (93.0)	1.1 (0.15–7.64)	
Parity (n = 1503)			
None	3 (101.4)	3.0 (0.95–9.17)	0.932
One	4 (163.6)	2.4 (0.92–6.51)	
Two	2 (94.7)	2.1 (0.53–8.45)	
Three or more	5 (131.4)	3.8 (1.58–9.14)	
Marital status (n = 1608)			
Married or cohabiting	1 (203.4)	0.01 (0.00–0.04)	0.018
None cohabiting couple	11 (247.3)	4.4 (2.46–8.03)	
Single, widowed or divorced	3 (74.1)	4.0 (1.31–12.55)	
Education (n = 1606)			
Primary or less	13 (281.2)	4.6 (2.68–7.96)	0.008
Secondary or more	2 (242.8)	0.824 (0.21–3.29)	
Religion (n = 1610)			
Christian	14 (468.4)	3.0 (1.77–5.05)	1.000
Muslim	0 (26.2)	0 (-)	
Other	1 (30.9)	3.2 (0.46–22.94)	
Occupation (n = 1598)			
Housewife	7 (141.8)	4.9 (2.35–10.35)	0.362
Farmer	6 (189.4)	3.2 (1.42–7.05)	
Trader	2 (89.0)	2.2 (0.56–8.98)	
Civil servant	0 (53.3)	0 (-)	
Other	0 (48.2)	0 (-)	
Wealth score (n = 1610)			
Low SES 0–3	10 (189.9)	5.3 (2.83–9.79)	0.015
High SES 4–9	5 (335.6)	1.5 (0.62–3.58)	
Financial dependence (n = 1601)			
Complete or for the most part	12 (322.4)	3.7 (2.11–6.53)	0.185
Not at all or for some part	3 (199.5)	1.5 (0.49–4.66)	
Travel distance in minutes (n = 1463)			
Less than or equal to 30 minutes	8 (340.1)	2.4 (1.18–4.70)	0.540
More than 30 minutes	5 (137.1)	3.6 (1.52–8.76)	
Partner HIV status (n = 1607)			
Known negative	9 (414.7)	2.2 (1.13–4.17)	0.100
Known positive, known and not specified or unknown	6 (110.0)	5.5 (2.45–12.14)	
Partner age (n = 1597)			
14–24 years of age	3 (113.0)	2.7 (0.86–8.23)	0.386
25–34 years of age	10 (262.9)	3.8 (2.05–7.07)	
35–60 years of age	2 (145.1)	1.4 (0.35–5.51)	
Partner education (n = 1590)			
Primary or less	8 (214.4)	3.7 (1.87–7.46)	0.274
Secondary or more	6 (305.2)	2.0 (0.88–4.38)	
Partner occupation (n = 1602)			
Farmer	8 (157.3)	5.1 (2.54–10.17)	0.436
Trader	5 (141.0)	3.5 (1.48–8.52)	
Civil servant	0 (61.1)	0 (-)	
Driver	0 (49.3)	0 (-)	
Armed forces	0 (14.7)	0 (-)	
Trucker	0 (9.6)	0 (-)	
Other	2 (90.1)	2.2 (0.56–8.88)	
HIV status ever discussed among the couple (n = 1603)			
Yes	8 (378.7)	2.1 (1.06–4.23)	0.139
No	7 (144.6)	4.8 (2.31–10.15)	
Partner joined ANC during this pregnancy (n = 1600)			
Yes	6 (235.4)	2.5 (1.15–5.67)	1.000
No	8 (287.2)	2.8 (1.39–5.57)	
Couple ever tested for HIV jointly (n = 1602)			
Yes	5 (285.6)	1.8 (0.73–4.21)	0.180
No	9 (237.7)	3.8 (1.97–7.28)	
Partner circumcised (n = 1606)			
Yes	7 (317.4)	2.2 (1.05–4.63)	0.293
No	8 (207.1)	3.9 (1.93–7.73)	
Own HIV risk perception (n = 1608)			
High	5 (44.8)	11.2 (4.64–26.79)	0.003
Some	5 (138.2)	3.6 (1.51–8.69)	
Very low	2 (126.8)	1.6 (0.40–6.31)	
Not at risk	2 (215.0)	0.9 (0.23–3.72)	
Sexually active in this pregnancy (n = 1604)			
Yes	12 (374.2)	3.2 (1.82–5.65)	0.578
No	3 (149.6)	2.0 (0.65–6.22)	
Average sexual acts per month (n = 1034)			
4 or less	7 (156.9)	4.5 (2.13–9.36)	0.363
4–9	1 (88.2)	1.1 (0.16–8.05)	
10 or more	4 (88.9)	4.5 (1.69–11.99)	
Number of sexual partners in the past year (n = 1578)			
One or less	8 (439.8)	1.8 (0.91–3.64)	0.009
Two	3 (52.9)	5.7 (1.83–17.59)	
Three or more	3 (23.8)	12.6 (4.07–39.11)	
Number of sexual partners in this pregnancy (n = 1568)			
None	0 (8.6)	0 (-)	0.001
One	11 (498.1)	2.2 (1.22–3.99)	
Two or more	3 (6.2)	48.1 (15.53–149.27)	
If sexually active in this pregnancy, use condom (n = 1571)			
Always	0 (7.1)	0 (-)	0.823
Sometimes	2 (50.5)	4.0 (0.99–15.84)	
Rarely	1 (47.6)	2.1 (0.30–14.92)	
Never	12 (407.8)	2.9 (1.67–5.18)	
Unprotected sex with unknown person (n = 1610)			
Yes	6 (14.8)	40.6 (18.22–90.27)	< 0.001
No	9 (510.7)	1.8 (0.92–3.39)	
Alcohol abuse (n = 1610)			
Yes	7 (29.4)	23.8 (11.35–49.93)	< 0.001
No	8 (496.1)	1.6 (0.81–32.24)	
Sex under influence of drugs or alcohol (n = 1610)			
Yes	2 (5.7)	35.0 (8.76–140.09)	0.010
No	13 (519.8)	2.5 (1.45–43.07)	
STD^1^ during pregnancy (n = 1600)			
Yes	2 (60.4)	3.3 (0.83–13.25)	0.681
No	13 (462.4)	2.8 (1.63–4.84)	
Mental health condition^2^ (n = 1597)			
Yes	1 (3.4)	29.2 (4.11–207.10)	0.099
No	14 (517.8)	2.7 (1.60–4.57)	
Do you ever discuss condom use with the partner? (n = 1608)			
Yes	5 (176.0)	2.8 (1.18–6.83)	0.615
No	10 (348.8)	2.9 (1.54–5.33)	
Partner agrees to condom use when asked (n = 1610)			
Always	0 (18.8)	0 (-)	1.000
Mostly	1 (42.3)	2.4 (0.33–16.80)	
Rarely	3 (103.5)	2.9 (0.09–8.99)	
Never	11 (357.8)	3.1 (1.70–5.55)	
Does partner have other sexual relations? (n = 1600)			
Yes	6 (97.1)	6.2 (2.78–13.75)	0.146
No	6 (276.9)	2.2 (0.97–4.82)	
I don’t know	3 (150.4)	2.0 (0.64–6.18)	
Partner often abroad or gone from home? (n = 1603)			
Yes	6 (169.9)	3.5 (1.59–7.86)	0.586
No	9 (353.6)	2.5 (1.32–4.89)	
Partner ever abusing alcohol^3^? (n = 1610)			
Yes	4 (100.1)	4.0 (1.50–10.65)	0.507
No	11 (425.5)	2.6 (1.43–4.67)	
Physical violence^4^ (n = 1599)			
Yes	3 (47.8)	6.3 (2.03–19.46)	0.149
No	12 (474.5)	2.5 (1.44–4.45)	

^1^Sexually transmitted diseases.

^2^Defined as a mental condition the woman received treatment for.

^3^Defined as excessive alcohol drinking several days a week.

^4^Defined as the experience of the partner beating, slapping, shoving, kicking, holding against her will or otherwise physically hurting or violating the woman.

In multivariable analysis using two different regression models, marital status remained a significant influencing factor. (Table 3) Women in a relationship, but living separately from their partners, were at higher risk of acquiring HIV during pregnancy compared to those married or living with their partner (OR 6.2, p-value 0.037 and IRR 8.78, p-value 0.038). Participants with higher education had presented lower risk compared to those who were less educated. However, these differences were only tendencies with borderline significance (OR 0.27, p-value 0.067 and IRR 0.23, p-value 0.061). In contradiction to the results obtained using Fisher’s exact test, no significant association was found between SES of participants and HIV seroconversion in pregnancy (OR 0.45, p-value 0.142 and IRR 0.43, p-value 0.139).

**Table 3:** Association of socio-demographic characteristics with HIV incidence during pregnancy.

Variable	Model 1: Penalized maximum likelihood logistic regression ^a^ (n = 1595 ^c^)	Model 2: Poisson regression ^b^ (n = 1595 ^c^)
	HIV incidence during pregnancy	
	OR (95% CI), P-value	IRR (95% CI), P-value
Age ^d^	0.98 (0.89–1.07), 0.645	0.97 (0.89–1.07), 0.578
Marital status ^d^		
Married or cohabiting (reference)	-	-
None cohabiting couple	6.22 (1.12–34.50), 0.037	8.78 (1.13–68.33), 0.038
Single, widowed or divorced	4.43 (0.59–33.10), 0.147	5.64 (0.55–58.38), 0.147
Education ^d^		
Primary or less (reference)	-	-
Secondary or more	0.27 (0.07–1.10), 0.067	0.23 (0.05–1.07), 0.061
Wealth score ^d^		
Low SES 0–3 (reference)	-	-
High SES 4–9	0.45 (0.15–1.31), 0.142	0.43 (0.14–1.31), 0.139
Partner HIV status ^d^		
Known negative (reference)	-	-
Known positive, known and not specified or unknown	1.90 (0.67–5.40), 0.231	1.82 (0.63–5.29), 0.270
Wald Chi-squared, P-value	14.86, 0.0214	-
LR Chi-squared, P-value	-	20.79, 0.0020
Deviance goodness of fit, P-value	-	119.20, 1.0000 ^f^
Pearson goodness of fit, P-value	-	1661.09, 0.0987 ^f^

^a^ Penalized maximum likelihood logistic regression is intended for rare events.

^b^ Poisson regression is intended for comparing rates of rare events.

^c^ Observations with missing values were excluded for all categorical or binary variables in the two regression models.

^d^ The two models are showing matching results.

^f^ P-value is showing the model to be a good fit.

OR, Odds ratio; CI, Confidence interval; IRR, Incidence rate ratio.

### Association of sexual risk behaviors with HIV incidence during pregnancy

Fisher’s exact test revealed significant associations of HIV seroconversion with certain behaviors including the number of sexual partners in the past year as well as in this pregnancy (p-value 0.009 and 0.001 respectively), unprotected sex with an unknown person or persons (p-value <0.001), as well as alcohol abuse and having sex under the influence of drugs or alcohol (p-value <0.001 and 0.010 respectively). Participants’ own HIV risk perception was associated with HIV incidence during pregnancy (p-value 0.003). (Table 2)

The results of the two regression models (Table 4) that were used to test for the association of risk behaviors with HIV seroconversion in pregnancy were in general in accordance with those obtained from univariable analysis. The risk of HIV infection during pregnancy was found to increase significantly with the number of sexual partners that participants had had in the past year (OR 1.26, p-value 0.006 and IRR 1.24, p-value 0.008) and in this pregnancy (OR 3.01, p-value 0.008 and IRR 2.78, p-value 0.008). Unprotected sex with an unknown person was also found to increase the risk of HIV acquisition in pregnancy (OR 16.74, p-value <0.001 and IRR 14.25, p-value <0.001). Likewise, alcohol abuse (OR 13.47, p-value <0.001 and IRR 12.08, p-value <0.001) as well as sex under the influence of alcohol or drugs (OR 8.30, p-value 0.006 and IRR 6.33, p-value 0.019) were identified as independent risk factors for HIV seroconversion in pregnancy.

On the other hand, the participants’ own perception of their HIV risk was found to be relevant only for those who perceived themselves to be ‘not at risk’, (OR 0.14, p-value 0.017 and IRR 0.13, p-value 0.021).

**Table 4:** Association of sexual risk behaviors with HIV incidence during pregnancy.

Variable (n) ^b^	Model 1: Adjusted penalized maximum likelihood logistic regression ^a^	Model 2: Adjusted Poisson regression ^a^
	HIV incidence during pregnancy	
	OR (95% CI), P-value	IRR (95% CI), P-value
Partner circumcised (n = 1592) ^c^		
No (reference)	-	-
Yes	0.75 (0.27–2.11), 0.587	0.75 (0.27–2.14), 0.589
Own HIV risk perception (n = 1593) ^c^		
High (reference)	-	-
Some	0.34 (0.10–1.18), 0.088	0.35 (0.10–1.24), 0.104
Very low	0.23 (0.05–1.11), 0.067	0.21 (0.40–1.15), 0.072
Not at risk	0.14 (0.03–0.71), 0.017	0.13 (0.02–0.74), 0.021
Sexually active in this pregnancy (n = 1590) ^c^		
No (reference)	-	-
Yes	1.76 (0.50–6.17), 0.376	1.95 (0.52–7.25), 0.321
Number of sexual partners in the past year (n = 1564) ^c^	1.26 (1.07–1.49), 0.006	1.24 (1.06–1.45), 0.008
Number of sexual partners in this pregnancy (n = 1556) ^c^	3.01 (1.33–6.81), 0.008	2.78 (1.30–5.94), 0.008
Unprotected sex with unknown person (n = 1556) ^c^		
No (reference)	-	-
Yes	16.74 (5.16–54.29), < 0.001	14.25 (4.52–44.93), < 0.001
Alcohol abuse (n = 1595) ^c^		
No (reference)	-	-
Yes	13.47 (4.60–39.45), < 0.001	12.08 (4.18–34.90), < 0.001
Sex under influence of drugs or alcohol (n = 1595) ^c^		
No (reference)	-	-
Yes	8.30 (1.83–36.66), 0.006	6.33 (1.36–29.47), 0.019
STD during pregnancy (n = 1586) ^c^		
No (reference)	-	-
Yes	1.28 (0.33–5.05), 0.723	1.08 (0.24–4.82), 0.917
Physical violence (n = 1585) ^c^		
No (reference)	-	-
Yes	1.96 (0.57–6.76), 0.290	1.74 (0.48–6.37), 0.403

^a^ Both regression models were adjusted for socio-demographic characteristics including: age, marital status, education, wealth score and partner HIV status.

^b^ n refers here to the total number of observations included in each of the two regression models.

^c^ The two models are showing matching results.

OR, Odds ratio; CI, Confidence interval; IRR, Incidence rate ratio.

## Discussion

Our study revealed an overall HIV incidence rate of 2.9/100 person-years among pregnant women in Kabarole District. A recent study in Rakai, Uganda from 2016 reported an HIV incidence rate of 0.84/100 person-years among the general female population of the same age group (15–49 years of age) [15]. Compared to our results, this suggests there might be a threefold higher HIV incidence rate during pregnancy. Previous studies have shown a similar trend [6,16,17], attributing it to a combination of pregnancy-related biological and behavioral factors including alterations of the genital tract mucosa, increased susceptibility to STDs, and a lower rate of condom use [17,18].

The HIV incidence rate in pregnancy in our study corresponds to findings from Uganda (2.3/100 person-years; 2005) [6] and from Kenya (2.31/100 person-years; 2015) [17]. However, it was lower than identified rates in Cameroon in 2016 (6.8/100 person-years) [19] and in South Africa in 2009 (10.7/100 person-years) [16].

Women in a relationship, but living separately from their partners, were at ten times higher risk of seroconverting during pregnancy compared to those living in the same household with their partner. Similarly, though only showing borderline significance, women with a primary education level or less were at higher risk compared to those with at least a secondary education. Previous studies indicated similar socio-demographic trends, with higher HIV incidence in pregnancy among women with less education [16] and among unmarried, single, or divorced women, as compared to married women [16,19].

We found no significant association between partner HIV status and HIV incidence among participants in pregnancy. On the other hand, participants reporting unprotected sex with an unknown person or a greater number of sexual partners were at significantly higher risk of HIV acquisition. This suggests that participants might have acquired new HIV infection through an unprotected sexual relationship with an external HIV-seropositive partner, rather than in the course of a committed relationship due to a pregnancy-associated increase in biological susceptibility to HIV. Previous studies have also suggested that women in a relationship with a known HIV-infected partner may be at lower risk compared to those who have a partner with unknown HIV status. This was mainly attributed to increased awareness through repetitive counselling, reinforced prevention measures, and low viral loads due to widespread antiretroviral drug coverage [5,17]. Therefore, male partner involvement in antenatal care with simultaneous HIV testing remains crucial for the prevention of HIV-infections during pregnancy [17,18].

Alcohol abuse and sex under the influence of alcohol or drugs were identified as risk factors for HIV seroconversion among pregnant women in our study. This is among the first studies to point out a significant association between alcohol abuse and HIV incidence in pregnancy. Nonetheless, the association between alcohol consumption and the incidence of HIV in the general population has been thoroughly established [20–22]. Alcohol seems to increase the risk of HIV by a number of pathways, including the influence of alcohol on personal behavior in general and on sexual conduct in particular. In addition, some studies have suggested a potential biological influence of alcohol, which can compromise the functions of the liver and the immune system and may therefore increase susceptibility to HIV [23–25]. On the other hand, studies have suggested that this association of HIV infection and alcohol could be confounded through personal traits and psychological disorders simultaneously associated with alcohol abuse and risky sexual behavior [20,26,27].

Almost 6% of the women in our study acknowledged drinking alcohol during pregnancy. Alcohol consumption while pregnant can lead to foetal alcohol syndrome and drastic life-long consequences for the unborn child, including behavioral disorders, mental retardation, and microcephaly. Therefore, ANC counselling should put emphasis on completely abstaining from alcohol consumption during pregnancy, while women with alcohol use disorder or alcohol dependency should be offered specific medical support and treatment.

Several studies have shown an association between physical or gender-based violence and HIV incidence [28–30]. While we could not find a significant association between physical violence and the incidence of HIV among participants in pregnancy, only 9% of the women in our study reported having been victims of physical violence. In comparison, data on gender-based violence shows a prevalence of 33% among women of the same age group in Uganda [31]. This may suggest vast underreporting in our study. Furthermore, we cannot preclude that some reported behaviors, like unprotected sex with unknown persons or having multiple sexual partners, could also stand for actual exposure to sexual or physical violence.

Likewise, we found no significant association between sexually transmitted diseases (STDs) and HIV incidence in pregnancy. This result is in contradiction to other studies, which have shown that women with STDs including syphilis, chlamydia, yeast infections, gonorrhoea, and trichomoniasis were at significantly higher risk of acquiring HIV in pregnancy [17,32]. However, no laboratory diagnosis was provided in our study, which may have led to false negatives.

The high HIV incidence among pregnant women in Kabarole District, Uganda emphasizes the importance of retesting for HIV during pregnancy. This has already been recommended by WHO as an essential component of PMTCT. Current guidelines recommend testing women as soon as they enter ANC, and retesting pregnant women in the third trimester, during delivery or shortly thereafter [33]; however, it remains unclear whether these practices are implemented systematically in most health facilities given that many of them have limited resources and often face overcrowded ANC wards and staff shortages.

Another important component of a comprehensive HIV prevention strategy is the early identification of pregnant women´s exposure to known risk factors and routinely offering them pre-exposure prophylaxis (PrEP) to avoid seroconversion. Current WHO guidelines recommend offering PrEP to individuals rather than to specific subgroups at high risk. A substantial risk of acquiring HIV in this context is defined as an incidence rate higher than 3/100 person-years, which applies to two of our study sites. Even though PrEP has been proven safe in early pregnancy, WHO is recommending further research on this prevention strategy among pregnant women [34–37]. At the time of the study, PreP was only available in specific pilot projects in the country, but Uganda introduced the provision of PreP for high-risk individuals into the National Guidelines on HIV prevention and treatment in 2018 [38,39]. As of this writing, however, these guidelines do not provide for any specific screening of the target group of pregnant women for PrEP.

The study had certain limitations. No randomized cluster sampling was adopted. This might have led to a selection bias making the study sample less representative. In addition, data collection was carried out using an interview-based questionnaire that included questions on sensitive topics. This could have led to social desirability bias; however, study staff was trained to ask and probe statements considerately and respectfully, and the interviews were conducted in a confidential setting and atmosphere. These policies were adopted in order to reduce biased answers as much as possible. Partner HIV status was not actively tested; we depended on the participants’ answers instead. Finally, even though the detected incidence rate was quite high, the total number of HIV seroconversions among participants was relatively small (only 15 cases) limiting the power of the study and making it less likely to establish significant associations.

## Conclusions

In spite of the existing efforts towards HIV prevention in Uganda, pregnant women are at considerably higher risk of acquiring HIV than the general population. Incidence rates in our study were intolerably high, especially in the rural setting. The study has identified certain socio-demographic and behavioral risk factors for contracting HIV during pregnancy, including women in non-cohabiting relationships, alcohol abuse, sex under the influence of drugs or alcohol, having several sexual partners and unprotected sex with unknown persons. Health service implementers should strongly reinforce HIV testing for all women as soon as possible in pregnancy, as well as scheduling retesting in the third trimester or around delivery. They should also encourage male partner involvement in ANC with simultaneous partner HIV testing. Ultimately, PrEP should be offered to individuals who are identified as being at high risk of infection, so as to protect mothers and their unborn children.

## Supporting information

S1 Data (PDF)

S2 Data (PDF)
