# Supplementary material for: The incidence of HIV and associated risk factors among pregnant women in Kabarole District, Uganda
Source: PLoS One. 2020 Jun 5;15(6):e0234174. doi: 10.1371/journal.pone.0234174 (PMC7274402; doi:10.1371/journal.pone.0234174)
Supplement: S1 Data — (PDF) [file pone.0234174.s001.pdf]

HIV incidence in pregnancy, Fort Portal- Study Questionnaire

Hospital registration Number ___________________________Date of visit___________________________________

Study ID____Staff name_____________________________________

Client Information

1. Client Type: **ANC client** □ **If ANC client,** gestational week: __________ **ANC visit no:** ___________

OR **Delivery client** □ **If delivery client,** date of delivery: __________________________________

2. Age (years):_________________ **3.Gravida:** _________________ **4.Para:** ___________________________

5. Date last HIV test BEFORE repeat test: _____________________ **/Gest. week**:____ **Confirm test was negative:** □

6. Marital status: Married/Cohabiting couple □ Not cohabiting couple □ Single □ Widowed □ Divorced □

7. Completed education: None □ Primary □ Secondary □ Tertiary □

8. Occupation: Housewife □ Farmer □ Trader □ Civil Service □ Other □:______________________________

9. Religion: ___________________________ **10. Residence:** Fort Portal □ Other □, specify: ____________________

11. Travel distance Hospital minutes: __________________**12.Travel cost** (one-way,UGX): ____________________

13. No. of people living in household: _________________**14. No. children <15 in household:** _________________

15. Is the following present in the household:

Pipe borne water	Yes □	No □	Electricity	Yes □	No □
Radio	Yes □	No □	Cupboard	Yes □	No □
Fridge	Yes □	No □	TV	Yes □	No □
Motor-bike, Car	Yes □	No □	Cattle	Yes □	No □
			Mosquito- net	Yes □	No □

Partner Information (applies for husband/partner, or father of this baby)

16. Age (years):_____________________ **17. Partnership duration** in years/months: _________________________

18. Completed education: None □ Primary □ Secondary □ Tertiary □

19. Occupation: Farmer □ Fisherfolk □ Trader □ Civil Service □ Trucker □ Armed Forces □

Other □, specify: ____

20. Religion: _____________________ **21. Residence:** Fort Portal □ Other □, specify: ________________________

22. HIV status of partner known to woman □ unknown to woman □ **If known:** positive □ negative □

23. When was partner tested for HIV the last time? ____________________________________ unknown □

24. Was HIV testing or status ever discussed in between the couple? Yes □ No □

25. Has your partner ever accompanied you to ANC during this pregnancy? Yes □ No □

If yes, was joint HIV partner testing performed in ANC during this pregnancy? Yes □ No □

26. Was HIV testing ever done jointly? Yes □, when? ______________________________________ No □

27. Is your partner circumcised? Yes □, since: _____________________________________ No □

Behavioral information, client

28. Do you think you might be at risk of getting HIV? High risk □ Some risk □ Very low risk □ Not at risk □

29. Have you been sexually active in this pregnancy? Yes □: average no. sexual acts per month _________ No □

If yes, how often did you use a condom? Always □ Sometimes □ Rarely □ Never □

If not always, please state reasons for not using condoms: ______________________________

30. Do you know how to use condoms? Yes □ No □ **31. Do you know where to get condoms?** Yes □ No □

32. How many sexual partners did you have: in the past year? _________During this pregnancy? ____________

33. In the past year, have you engaged in any of the following:

Intravenous drug use □ Commercial sex work □ Unprotected sex with someone not known well □

Trading sex for food or other goods or benefits □ Alcohol abuse □ Sex under influence of alcohol/drugs □

Sex with someone who was under influence of alcohol/drugs □ Sex involving anal intercourse □

If yes for any of these, please specify when/how often: ______________________________

34. Are you aware of any sexually transmitted disease (STD) you have had during this pregnancy?

No □ Yes □, please specify symptoms or diagnosis: ______________________________

If yes, have you received medical care for the disease? Yes □ No □

35. Do you suffer from any mental health condition? No □ Yes □, specify: ______________________________

Behavioral information, partner (applies for husband/partner, or father of this baby)

36. Do you ever discuss condom use with your partner? Yes □ No □, why not? ______________________________

37. Does your partner agree to use a condom when you ask him to? Always □ Mostly □ Rarely □ Never □

38. Do you generally consider your partner as being faithful? Yes □ No □ Don´t know □

39. Are you aware of other sexual relationships your partner may have? Yes □ No □ Don´t know □

40. Does your partner spend much time abroad or away from home? Yes □ No □

If yes, please specify reason and frequency: ______________________________

41. To your knowledge, does your partner ever abuse alcohol □ use intravenous drugs □ visit sex workers □

If yes, please estimate frequency of events______________________________

42. Have you ever experienced any physical violence by your partner? Yes □ No □

If yes, please specify frequency and event: ______________________________

43. Has your partner ever repressed/threatened you emotionally □ financially (e.g. depriving of money) □

If yes, specify: ______________________________

44. Are you financially dependent on your partner? Completely□ For the most part□ For some part□ Not at all□

Result of client´s HIV repeat test

Repeat test performed during this session today □ Result was positive □ Result was negative □

OR (only if a repeat HIV test was performed already, at a time less than 3 months ago)

Result transferred from hospital records □ Date of repeat test ____________________/Gest. week___________

Test result of transferred hospital records: Result was positive □ Result was negative □
