# Supplementary material for: The incidence of HIV and associated risk factors among pregnant women in Kabarole District, Uganda
Source: PLoS One. 2020 Jun 5;15(6):e0234174. doi: 10.1371/journal.pone.0234174 (PMC7274402; doi:10.1371/journal.pone.0234174)
Supplement: S2 Data — (PDF) [file pone.0234174.s002.pdf]

Study questionnaire-Rutooro version

OKUKAGUZIBWAHO HAKAHUKA KOMUNYWERERRO OMUBAKYARA ABAINE ENDA OMURUBUGA RWA FORT PORTAL- UGANDA

Enamba yawe hairwarro linu ____________________________Ebiro byokwehandikisa___________________________

Enamba yawe habwokuseruliriza kunu_____________________________

Ibara Lyogu arukukukaguza____

Ibara Lyawe ____

Ebirukukwata hakukebeza enda

Akehandikisa kwekebeza end hairwarro linu ? □ ego □ nangwa

Obukiraba ego, enda ina wiki zingaha: ________

Murundi gwakaingaha kukeberwa end kwiha nagitwara? __________________

Rundi mukazi aizire kuzaara? □ ego □ nangwa obukiraba ego, ebirobyokuzarramu: ___________________

Emyaka yobukuru:__________________ Enda yakaingaha: __________________

Ebiro byokwezi yamalirire kukeberwa akahuka komunywererro kwiha natwara end: ___________________

Enda ekaba ina wiki zingaha:____ Akahuka komunywererro kali karumu rundi nangwa: □karumu □nangwa

Obuswezi: aswirwe □ wakacu □ aliwenka□ mufakati □ akahukanana □

Okusoma: atasome □ wa puraimare□ wa seniya □ akamara kosi □

Omulimo: mufumbo □ mulimi □ musubuzi □ mukozi wa gafumenti □ ebindi □**:**_______________

Edini: ________________________________ harukwikara: Fort Portal □ Other □, specify: _____________________

Nikitwara bwirreki kuhika hairwarro:_____________________

nikitwara sente zingaha kuhika hairwarro (UGS): _________________________________

Abantu aboine munju yawe:________________ abana abali hansi yemwaka 15 omunju yawe_________________

Binu obine munju yawe?:

Amasanyarazi	ego □	nangwa □
Amaizi ga tapu	ego □	nangwa □
Radio	ego □	nangwa □
TV	ego □	nangwa □
Friji	ego □	nangwa □
Ente	ego □	nangwa □
kabodi	ego □	nangwa □
boda/pikipiki	ego □	nangwa□
motoka	ego □	nangwa□
Akatimba kemibu	ego □	nangwa □

Obirukukwataho omugonzibwa wawe (ogu rwamutwekere enda)

Emyakaye:______________________ **Okasumi mwakamara hamu**: ________________________________

Okusoma kwe: atasome□ waprimare□ waseniya□ wa kosi □

Omulimo arukukora: mulimi/mulisa □ musohi□ musubuzi □ mukozi wagavumenti □ muvugi□ webyokwerinda □ Ebindi□, soborra hanu ____

Edini ye: __________________________ Harukwikara: Fort Portal □ Handi □, soborra: ___________________________

Nomanya omusaija wawe obu araba aine akahuka komunywererro? □ ego □ nangwa Obukiraba ngu ego: akaine □ nangw □

Omusaija wawe akamalirra di kwekebeza (ebiro byokwezi)? ______________________________________

tindikubimanya □

Mwarabalizeho ensonga yokwekebeza akahuka komunywererro? ego □ nangwa □

Omusaija wawe yarakusendekerizeho kakugenda mwirwarro kwekebeza akahuka komunywererro kwiha notwara enda? ego □ nangwa □

Ekiraba ego, inyena mukakeberwa akahuka komunywererro kwiha notwara enda enu? ego □ nangwa □

Obukiraba nangwa, mwarayekebizeho akahuka komunywererro? ego □ nangwa □

Obukiraba ego, mukakeberwa di (ebiro byokwezi) ? ____

Okwetwaza kwawe

Nuhurra oyeralikirire ngu nosobora kukwatwa akahuka komunywererro? nyeralikerire muno □ nyerlikirire kake □ tinyeralikirire □

Obaire noterana nomusija kwiha notwara enda enu? □ ego □ nangwa

Obukiraba ego, mukira kuterana emirundi ingaha buli mwezi: _________

Mukuterna nomusaija mukozesa Kondomu ? □ ego □ nangwa

Obukira ego, kondomu mukira kuzikozesa di? Buli kuterana □ dimunadimu □ titukira kuzikozesa □

Obukira nangwa, soborraho ensenga habwaki:

____

Nomanya kukozesa kondomu? ego □ nangwa □ **Nomanya hokwiha kondom?** ego□ nangwa □

Wakaterana nbasaija baingaha: kiwiha omyaka nigutandika ? ____________ kwiha notwara end? ______________

Kwiha omwaka nigutandika, okabaho omubikorwa nkabinu:

Kwetera emibazi yebitokoza obwongo □ okwetunda □ kuterana nousaija owotamanyire obwaraba aine akahuka komunywererro □ okunywa ebitamiza nka amarwa □ kuterana amazire kutamira amarwa/ebitokoza bwongo □ kuterana nomusaoja atamire amarwa/ebitokoza bwongo □ kuterana nomusaija omulingo gwebisiyaga □

Obukiraba ego, kibaberaho nkaha kandi hidi : ____

Warakwasirweho endwaire yona eyeruraba mukuterana kwiha notwara enda enu?

ego □ nangwa□

Obukiraba ego, soborraho obwokurorraho bwendwaire egyo:

____

Obukiraba ego, okatungaho obujanjabi bwona omwirwarro? ego □ nangwa □

Warakwasirweho oburwaire byomutwe bwona? ego□ nangwa □

Obukiraba ego, soborraho endwaire enu: ______________________________________

Kwetwaza kwomusaija wawe (applies for husband/partner, or father of this baby)

Result of client´s HIV repeat test

Repeat test performed during this session today □ Result was positive □ Result was negative □

OR (only if a repeat HIV test was performed already, at a time less than 3 months ago)

Result transferred from hospital records □ Date of repeat test _______________________/Gest. week____________

Test result of transferred hospital records: Result was positive □ Result was negative □

If yes, please specify reason and frequency: ____

To your knowledge, does your partner ever abuse alcohol □ use intravenous drugs □ visit sex workers □

If yes, please estimate frequency of events____

Have you ever experienced physical violence by your partner? Yes □ No □

If yes, please specify frequency and event: ____

Has your partner ever repressed/threatened you emotionally □ financially □

If yes, specify: ____

[illegible]
